# Early Repetitive Transcranial Magnetic Stimulation Exerts Neuroprotective Effects and Improves Motor Functions in Hemiparkinsonian Rats

**DOI:** 10.1155/2021/1763533

**Published:** 2021-12-27

**Authors:** Tsung-Hsun Hsieh, Xiao-Kuo He, Hui-Hua Liu, Jia-Jin J. Chen, Chih-Wei Peng, Hao-Li Liu, Alexander Rotenberg, Ko-Ting Chen, Ming-Yuan Chang, Yung-Hsiao Chiang, Pi-Kai Chang, Chi-Wei Kuo

**Affiliations:** ^1^School of Physical Therapy and Graduate Institute of Rehabilitation Science, Chang Gung University, Taoyuan, Taiwan; ^2^Neuroscience Research Center, Chang Gung Memorial Hospital at Linkou, Taoyuan, Taiwan; ^3^Healthy Aging Research Center, Chang Gung University, Taoyuan, Taiwan; ^4^Department of Rehabilitation Medicine, Fifth Hospital of Xiamen, Xiamen, China; ^5^Department of Rehabilitation Medicine, Sun Yat-sen Memorial Hospital, Sun Yat-sen University, Guangzhou, China; ^6^Department of Biomedical Engineering, College of Engineering, National Cheng Kung University, Tainan, Taiwan; ^7^Medical Device Innovation Center, National Cheng Kung University, Tainan, Taiwan; ^8^School of Biomedical Engineering, College of Biomedical Engineering, Taipei Medical University, Taipei, Taiwan; ^9^Department of Electrical Engineering, National Taiwan University, Taipei, Taiwan; ^10^Department of Neurology, Boston Children's Hospital, Harvard Medical School, Boston, MA, USA; ^11^Department of Neurosurgery, Chang Gung Memorial Hospital at Linkou, Taoyuan, Taiwan; ^12^Division of Neurosurgery, Department of Surgery, Min-Sheng General Hospital, Taiwan; ^13^Department of Early Childhood and Family Educare, Chung Chou University of Science and Technology, Changhua County, Taiwan; ^14^Department of Surgery, School of Medicine, College of Medicine, Taipei Medical University, Taipei, Taiwan

## Abstract

Repetitive transcranial magnetic stimulation (rTMS) is a popular noninvasive technique for modulating motor cortical plasticity and has therapeutic potential for the treatment of Parkinson's disease (PD). However, the therapeutic benefits and related mechanisms of rTMS in PD are still uncertain. Accordingly, preclinical animal research is helpful for enabling translational research to explore an effective therapeutic strategy and for better understanding the underlying mechanisms. Therefore, the current study was designed to identify the therapeutic effects of rTMS on hemiparkinsonian rats. A hemiparkinsonian rat model, induced by unilateral injection of 6-hydroxydopamine (6-OHDA), was applied to evaluate the therapeutic potential of rTMS in motor functions and neuroprotective effect of dopaminergic neurons. Following early and long-term rTMS intervention with an intermittent theta burst stimulation (iTBS) paradigm (starting 24 h post-6-OHDA lesion, 1 session/day, 7 days/week, for a total of 4 weeks) in awake hemiparkinsonian rats, the effects of rTMS on the performance in detailed functional behavioral tests, including video-based gait analysis, the bar test for akinesia, apomorphine-induced rotational analysis, and tests of the degeneration level of dopaminergic neurons, were identified. We found that four weeks of rTMS intervention significantly reduced the aggravation of PD-related symptoms post-6-OHDA lesion. Immunohistochemically, the results showed that tyrosine hydroxylase- (TH-) positive neurons in the substantia nigra pars compacta (SNpc) and fibers in the striatum were significantly preserved in the rTMS treatment group. These findings suggest that early and long-term rTMS with the iTBS paradigm exerts neuroprotective effects and mitigates motor impairments in a hemiparkinsonian rat model. These results further highlight the potential therapeutic effects of rTMS and confirm that long-term rTMS treatment might have clinical relevance and usefulness as an additional treatment approach in individuals with PD.

## 1. Introduction

Parkinson's disease (PD) is recognized as the second most prevalent age-related neurodegenerative disorder after Alzheimer's disease, affecting approximately 1% of the population over the age of 60 years [1–4]. The major pathological hallmark of the disease results from the degeneration of dopaminergic cells in the substantia nigra *pars compacta* (SNpc), leading to several motor disturbances, e.g., tremor, muscular rigidity, bradykinesia, akinesia, and gait disturbance [5–8]. Currently, mainstream PD treatment is pharmacological management, such as dopamine supplementation (e.g., levodopa), dopamine agonists, catechol-O-methyltransferase inhibitors (COMTIs), or monoamine oxidase type B inhibitors (MAOBIs) [9–11]. Among them, the dopamine precursor levodopa is the most common and effective antiparkinsonian medicine and remains the mainstay of PD treatment [10–13]. However, with long-term dopaminergic replacement therapy, several complications, mainly motor in nature, such as motor fluctuations, levodopa-induced dyskinesia, freezing, gait disturbance, and postural instability, are common side effects following long-term administration of levodopa [10, 14, 15]. Consequently, a number of alternative nonpharmacological approaches, e.g., deep-brain stimulation (DBS), have been investigated as new therapeutic strategies for PD [16–19]. However, DBS, a stimulation technique that involves implanting electrodes deeply into a selected portion of the basal ganglia, requires an invasive stereotactic approach with intraparenchymal implantation and is a high-cost procedure [20–22]. Furthermore, although DBS may improve some PD symptoms, it cannot modulate disease progression [23, 24]. Therefore, an alternative and better treatment that can modify disease progression is urgently needed for PD.

Recent research suggests that noninvasive repetitive transcranial magnetic stimulation (rTMS) can modulate cortical excitability in the motor cortex via plasticity-like mechanisms [25–27]. Furthermore, the recently developed theta burst stimulation (TBS) scheme of rTMS is capable of modulating motor cortical excitability beyond a short period of stimulation (20-190 sec) and with lower stimulus intensity than conventional low- or high-frequency rTMS [28–30]. Thus, this rTMS-TBS protocol has been considered to have therapeutic potential for PD [31–33]. However, the results among various studies exploring the therapeutic effects of rTMS using the TBS protocol on PD have been inconsistent [34]. Improvements in motor function, such as gross movements of the hand and Unified Parkinson's Disease Rating Scale (UPDRS) motor subscores, in PD after rTMS using TBS have been reported [35]. Conversely, other studies performed in PD patients showed no improvements in gait, bradykinesia, UPDRS scores, or gait freezing using intermittent TBS [33, 36]. Although the exact underlying therapeutic mechanism is still unclear, the controversial results might be due to methodological differences, the heterogeneity of clinical presentations and disease severity, long-lasting pharmacological effects, and protocol variability [37–40].

Animal models of disease may help in exploring the effectiveness and developing the therapeutic strategy of rTMS protocols by eliminating the discrepancy to provide a more stable disease condition [41–43]. A few animal studies found that 4 weeks of low-frequency intervention (500 pulses at 0.5 Hz) or high-frequency (10 Hz for 20 min) rTMS improved locomotor functions, dopaminergic neuron survival, and rotational behavior in a midstage 6-hydroxydopamine- (6-OHDA-) induced hemiparkinsonian rat model [43, 44]. Moreover, based on our previous finding, rTMS-induced motor plasticity was reduced in 6-OHDA-induced hemiparkinsonian rats with advanced disease. Such a reduction in motor plasticity is strongly correlated with dopaminergic cell loss in the substantia nigra and the severity of PD symptoms [45]. However, it remains unclear whether earlier intervention with rTMS using the TBS protocol could lead to improved therapeutic effects on motor function and improved induction of a neuroprotective effect in dopaminergic neurons. Therefore, in the current study, a series of experiments was conducted to test the therapeutic potential of rTMS in a preclinical PD animal model as an early step toward eventual clinical application. We employed our previously developed quantitative motor performance assessment techniques to enhance the understanding of the underlying neuromodulation mechanisms. The effects of rTMS were mainly assessed by behavioral measurements and immunohistochemical analyses, including comprehensive video-based gait analysis, the bar test for akinesia, apomorphine-induced rotational analysis, and tests of the dopaminergic neuron degeneration level. It was hypothesized that long-term rTMS treatment, especially using the TBS protocol, would result in a lasting reduction in motor deficits and have a neuroprotective effect on dopaminergic neurons in 6-OHDA-induced hemiparkinsonian rats. The knowledge obtained in these experiments may have translational relevance for establishing new clinical therapeutic applications of rTMS.

## 2. Materials and Methods

### 2.1. Animals

Adult male Wistar rats (350-400 g; *N* = 41) obtained from the Animal Center of Chang Gung University were used for the present study. All rats were housed in a temperature-controlled animal care facility at a temperature of 25°C with a 12 h light/dark cycle. All experimental protocols and surgical procedures were approved and followed the guidelines of the Institutional Animal Care and Use Committee at Chang Gung University (IACUC Approval No. CGU16-031, with validation period 08/01/2016-07/31/2019). In the present study, all efforts were made to minimize animal suffering and the number of animals used.

### 2.2. Hemiparkinsonian Rat Model

The procedures for the induction of hemiparkinsonian rats were described previously [45–48]. Briefly, animals were deeply anesthetized by Zoletil (50 mg/kg, i.p.; Vibac Laboratories, France) and xylazine (10 mg/kg, Rompun, Bayer, Germany) and then mounted in a stereotaxic apparatus (Stoelting, Wood Dale, IL, USA). A 2 cm incision was made along the midline of the scalp, and the area was carefully cleared to expose the line of bregma. A small hole was drilled in the skull 4.3 mm posterior and 1.6 mm lateral (left side only) to the midline. A solution of 6-OHDA (8 *μ*g dissolved in 4 *μ*l 0.02% ascorbic saline) was injected into the left medial forebrain bundle (DV: 8.2 mm) at a rate of 0.5 *μ*l/min using a 10 *μ*l Hamilton microsyringe fitted with a 26-gauge steel cannula and mounted vertically on the stereotactic frame [49]. Before being retracted, the needle was left in the brain for 5 min to prevent backfilling along the injection tract [50]. To verify dopamine depletion after unilateral neurotoxin 6-OHDA infusion, a conventional and reliable apomorphine-induced rotation test was adopted [50–52]. In general, hemiparkinsonism induction by 6-OHDA was considered successful in rats with rotational responses of over 120 turns [53]. According to the criteria, at 4 weeks after surgery, all animals (*n* = 26) in both groups were regarded as hemiparkinsonian rats.

### 2.3. rTMS Treatment

**Figure 1:**
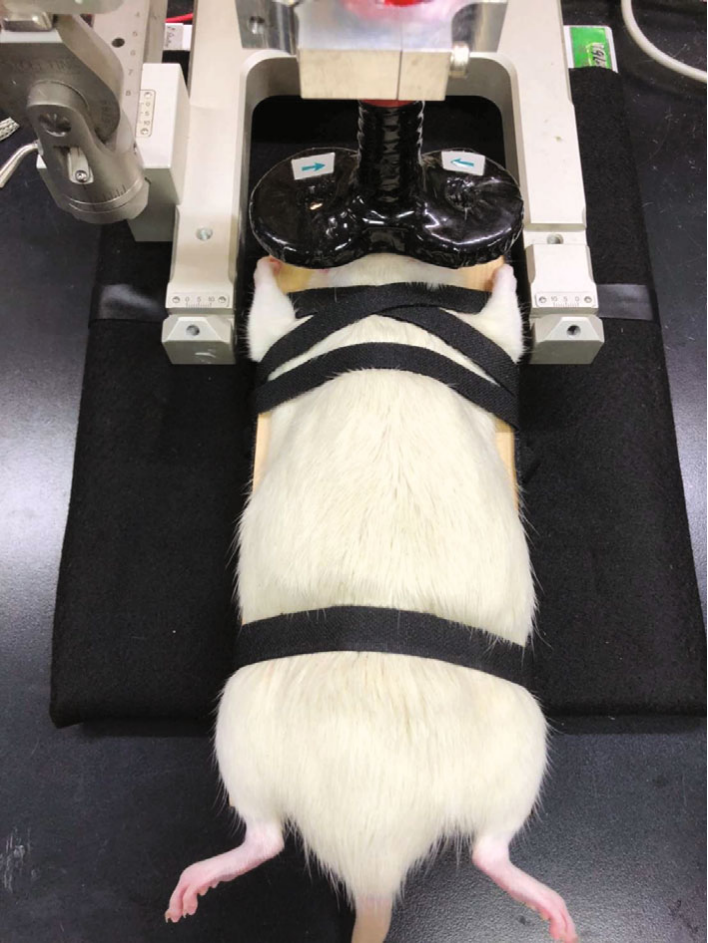
Setup of repetitive transcranial magnetic stimulation (rTMS) treatment for 6-OHDA-induced hemiparkinsonian rats. Unanesthetized rats were restrained on a platform with 4 straps with minimal discomfort. The figure-8 TMS coil is centered over the dorsal scalp at the interaural line to stimulate the bilateral motor cortex.

All rTMS treatments were performed using a figure-eight coil (25 mm double small coil, Magstim Co.) connected to a Rapid^2^ magnetic stimulator (Magstim Co., Whitland, Carmarthenshire, Wales, UK). To maintain the stability of the rTMS stimulation, the unanesthetized rats were restrained on a platform with four straps with minimal discomfort (Figure 1). The coil was held in the stereotaxic frame and positioned in the midline at the interocular line over the dorsal scalp, a position that can reliably and equally stimulate the bilateral motor cortex of limb and elicit bilateral limb movement [54]. In our experience, a rat can tolerate torso restraint for 5 min, which allows time for one session of TBS treatment [54]. The animals in the rTMS treatment group received the intermittent TBS (iTBS) paradigm (2 seconds of TBS training was repeated every 10 seconds for 20 repetitions for a total of 600 pulses each day for 7 consecutive days per week) under awake conditions for 4 weeks (28 consecutive sessions of iTBS in total) [45]. TBS consists of triplets of pulses at 50 Hz repeated every 200 ms [30]. The intensity of magnetic pulses was set at an 80% resting motor threshold, which was defined as the minimal intensity of magnetic stimulation required for eliciting minimal forelimb muscle twitches. The animals in the sham control group underwent an identical procedure to the experimental group except that rTMS-iTBS was replaced with sham rTMS with the magnetic coil placed 80 mm laterally and above the rat's head [55].

### 2.4. Behavioral Tests

A well-trained examiner was blinded to group assignment and performed all behavioral examinations before and after treatment. Three motor behavioral tests, i.e., video-based gait analysis, the bar test for akinesia, and an apomorphine-induced rotational behavior test, were performed in the same sequence on the same day to test the changes in functional motor performance in the sham rTMS (*n* = 13) and real rTMS groups (*n* = 13). There was at least a 2-hour break between tests to avoid possible interactions. The video-based gait analysis, bar test, and rotational behavior test were performed at baseline and after every weekly treatment.

#### 2.4.1. Video-Based Gait Analysis

To identify the changes in gait pattern in hemiparkinsonian rats with and without rTMS treatment, a walking track equipped with a video-based gait analysis system was applied to obtain the spatiotemporal parameters of the gait pattern. The procedure of video-based gait analysis to measure the gait pattern of hemiparkinsonian rats was described previously [46–48]. Briefly, the walking track equipment consisted of an enclosed walkway made of transparent Plexiglas (80 cm *L* × 6 cm *W* × 12 cm *H*) with a 45° tilting mirror positioned underneath the walkway. A high-speed and high-resolution camera (EX-F1, Casio, Japan) was positioned on the side of the walkway to capture the sagittal view and the reflected bottom view from the mirror. Before the experiment, all animals were acclimated to the walkway by allowing them to walk freely on the track for 20 min. Then, animals were trained to walk steadily on the Plexiglas walking track five times before formal recording. During the measurement, the rats were allowed to walk freely on the walking track at their own speed. The walking task was repeated in both directions for recording the movement of each hind limb. The walking task was repeated until five satisfactory walking trials were considered successful, meaning that at least four steps without pause were obtained during each test [46, 47]. The whole process for walking trials took approximately 30 minutes, and there was at least a 2-hour break between tests to prevent possible interactions. After recording, the image data captured from each trial were processed semiautomatically to identify the sequential footprints by MATLAB software (MathWorks, version 7.6., R2008a) [47]. Two spatial parameters (i.e., step length and stride length) and three temporal gait parameters (i.e., walking speed, stance phase time, and swing phase time) were determined in bilateral hind limbs of each group [46–48, 56].

#### 2.4.2. Bar Test

Akinesia is a typical symptom in PD. The bar test was adopted in this study to detect forelimb akinesia in hemiparkinsonian rats [46, 57]. During the bar test, each rat was placed gently on a table, and the affected forepaw (the paw contralateral to the 6-OHDA lesion) was placed on a horizontal acrylic bar (0.7 cm diameter), positioned 9 cm above the table surface. The duration of time (in sec) spent from placing the affected forepaw on the bar to the first complete removal from the support bar was recorded [46, 48]. The animals were subjected to five subsequent trials, which were video recorded, and the duration was averaged over these five trials.

#### 2.4.3. Apomorphine-Induced Spontaneous Rotation Test

Conventional and reliable apomorphine-induced contraversive rotational behavior was measured every week after the 6-OHDA injection to estimate the severity of dopamine depletion [46, 58, 59]. After the injection of apomorphine (0.5 mg/kg in 0.1% ascorbic acid, s.c.; Sigma), the hemiparkinsonian rats were placed in a round bowl (40 cm in diameter). Apomorphine-induced rotational behavior was recorded by a digital video camera for a 60 min period. For precise calculation of the number of rotations after apomorphine injection, the net number of rotations was manually calculated as the difference between the number of contralateral rotations and the number of ipsilateral rotations to the 6-OHDA lesion side (total right-total left 360° turns) after apomorphine injection from the 60-minute video recording.

### 2.5. Immunohistochemistry Investigation

After behavioral tests were performed on days 7 and 28 postlesion, animals were sacrificed for tyrosine hydroxylase (TH) staining. TH staining analysis was carried out according to a previously employed protocol [45, 48, 60]. Briefly, animals were deeply anesthetized with an overdose of pentobarbital (60 mg/kg i.p., Apoteksbolaget, Sweden) and perfused transcardially with phosphate-buffered saline (PBS) and 4% paraformaldehyde solution (PFA). Brains were carefully removed, postfixed for 3 days, and cryoprotected in 30% sucrose solution at 4°C until the brain sank. The brains were sectioned into coronal blocks at a thickness of 30 *μ*m on a cryostat (Leica CM3050 S Cryostat, FL, USA), and the areas of the SNpc and striatum were selected [48]. The free-floating sections were quenched with 0.3% H_2_O_2_/PBS for 10 min and 10% milk (ANCHOR SHAPE-UP, New Zealand) for 1 hour to block nonspecific antibody binding. Sections were then incubated with rabbit primary anti-TH (1 : 1000, AB152, Millipore, USA) for 1 hour at room temperature. Thereafter, sections were washed in PBS and incubated for 1 h with the secondary anti-rabbit antibody (1 : 200, MP-7401, Vector Labs, USA) in PBS. After rinsing, sections were placed in 3,3-diaminobenzidine (DAB, SK-4105, Vector Labs, USA) for 3-5 min. Finally, the sections were mounted on slides, dehydrated in a series of alcohols, cleared in xylene, and cover-slipped in DPX. The mounted coronal sections were digitally scanned at 40x magnification (0.25 *μ*M/pixel) using a digital pathology slide scanner (Aperio CS2, Leica Biosystems Inc. Buffalo Grove, IL, USA) and viewed with Aperio ImageScope software. The obtained images were converted into binary (8-bit black-and-white) images. The binary threshold was determined to capture the TH-positive cells in the regions of interest while minimizing background staining and was kept constant for all images. The numbers of TH-positive cells in each region were counted by means of particle analysis using computer-based image analysis software (Image-Pro Plus 6.0, Media Cybernetics, Bethesda, MD, USA), and these values were then manually validated by two investigators to ensure the correct identification of immunoreactivity patterns. The percentage loss of TH-positive cells was calculated in the ipsilateral hemisphere and normalized with respect to the contralateral side. With regard to the striatal TH-positive fibers, the optical density of TH-positive fibers in the striatal sections was analyzed using Image-Pro Plus 6.0 software (Media Cybernetics, USA) with correction for nonspecific background density measured at the corpus callosum. The percentage loss of dopaminergic fibers on the ipsilateral side was normalized and presented with respect to the contralateral side.

### 2.6. Experimental Design

**Figure 2:**
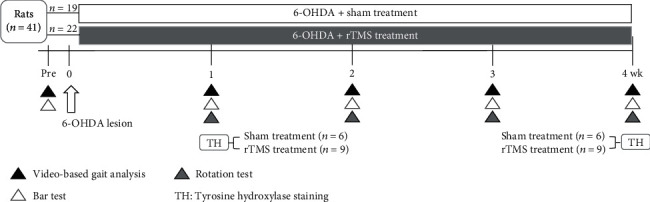
Design of the study of the long-term treatment effects of rTMS on rats with 6-OHDA-induced PD. rTMS and sham control treatments were performed daily over 4 successive weeks. Behavioral tests, including gait analysis, the bar test, and apomorphine-induced rotation tests, were performed every week to investigate the time-course treatment effects. Immunohistochemistry tests were performed at week 1 and week 4 post-6-OHDA lesion to identify the neuroprotective effects rTMS treatment on dopaminergic neurons and fibers.

To verify the therapeutic effects of long-term rTMS intervention in hemiparkinsonian rats, the experimental hemiparkinsonian rats were randomly divided into a 6-OHDA+rTMS treatment group (*n* = 22) and a 6-OHDA+sham treatment group (*n* = 19). For the early and long-term rTMS intervention, hemiparkinsonian rats were randomly assigned to receive sham or real rTMS intervention. Starting 24 h after 6-OHDA injection, neurotoxic PD rats received daily sham or real rTMS under awake conditions for 7 consecutive days/week for 4 weeks (Figure 2). Behavioral tests, including detailed video-based gait analysis and the bar test, were performed at baseline and every week after 6-OHDA was injected until the end of the rTMS intervention. Apomorphine-induced rotational behavior was measured every week after 6-OHDA through 4 weeks of rTMS treatment. Tyrosine hydroxylase (TH) staining was assessed in randomly selected hemiparkinsonian rats after behavioral tests at 1 week (*n* = 9 in the 6-OHDA+rTMS group; *n* = 6 in the 6-OHD+sham treatment group) and 4 weeks (*n* = 9 in the 6-OHDA+rTMS group; *n* = 6 in the 6-OHD+sham treatment group) post-6-OHDA lesion.

### 2.7. Data Analysis

#### 2.7.1. Statistical Analysis

Data were analyzed using SPSS version 17.0 with the significance level set as *P* < 0.05 for each assessment. All data are presented as the average ± standard error of the mean (SEM). The effect of rTMS on the behavioral test performance was evaluated by a two-way repeated-measures analysis of variance (ANOVA) with protocol (real vs. sham) as the between-subject factor and time (pre, every week during intervention) as the within-subject factor. No preintervention data were included for apomorphine-induced rotation in the early intervention session because there was no rotation before 6-OHDA was injected. Unpaired *t-*tests were performed to compare groups at each time point when the main effect of the group was significant. Furthermore, a separate one-way ANOVA followed by post hoc Fisher's LSD tests was used to compare behavioral and immunohistochemical data between time points when needed.

## 3. Results

### 3.1. Effect of rTMS Intervention on Behavioral Assessments

**Figure 3:**
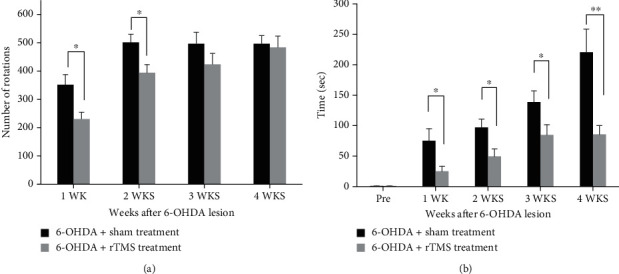
Time-course analysis of apomorphine-induced rotational behavior (a) and duration of bar test for akinesia (b) observed over 4 weeks in the sham- and rTMS-treated groups. Error bars = SEM; ^∗^*p* < 0.05 and ^∗∗^*p* < 0.01, significant difference between the two groups.

Performances in the detailed video-based gait analysis and bar test were assessed in the 6-OHDA+rTMS treatment group (*n* = 13) and 6-OHDA+sham treatment group (*n* = 13) at baseline and every week after the 6-OHDA lesion until the end of the rTMS intervention. The rotation test was performed every week after 6-OHDA until 4 weeks of rTMS treatment in both groups (*n* = 13 in each group). The descriptive and inferential statistics of the primary outcomes for all the behavioral tests are shown in Supplementary Table S1.  Figure 3(a) shows the time-course changes in the apomorphine-induced rotation response (net of contralateral rotations/hour) after lesion induction. Two-way repeated-measures ANOVA showed significant main effects in time (*F*_3,72_ = 18.94, *p* < 0.001) and in protocol (*F*_1,24_ = 5.875, *p* = 0.023). The post hoc *t*-tests between the two groups revealed that the rotation number reached significant differences at 1 week (*t* = 2.771; *p* = 0.011) and 2 weeks (*t* = 2.595; *p* = 0.016) posttreatment but not at 3 weeks (*t* = 1.300; *p* = 0.206) or 4 weeks post-6-OHDA lesion (*t* = 0.258; *p* < 0.799).  Figure 3(b) shows the time-course changes in the performance in the bar test for akinesia in the 6-OHDA+rTMS- and 6-OHDA+sham-treated rats over a 4-week observation period. Two-way repeated-measures ANOVA revealed significant effects of time (*F*_4,96_ = 24.879, *p* < 0.001) and group (*F*_1,24_ = 18.434, *p* < 0.001) on the contralateral (affected) limb. Subsequent post hoc *t*-tests between the groups showed that the bar test scores reached significant differences at 1 week (*t* = 2.341; *p* = 0.032), 2 weeks (*t* = 2.573; *p* = 0.017), 3 weeks (*t* = 2.182; *p* = 0.039), and 4 weeks post-6-OHDA lesion (*t* = 3.322; *p* = 0.004).

**Figure 4:**
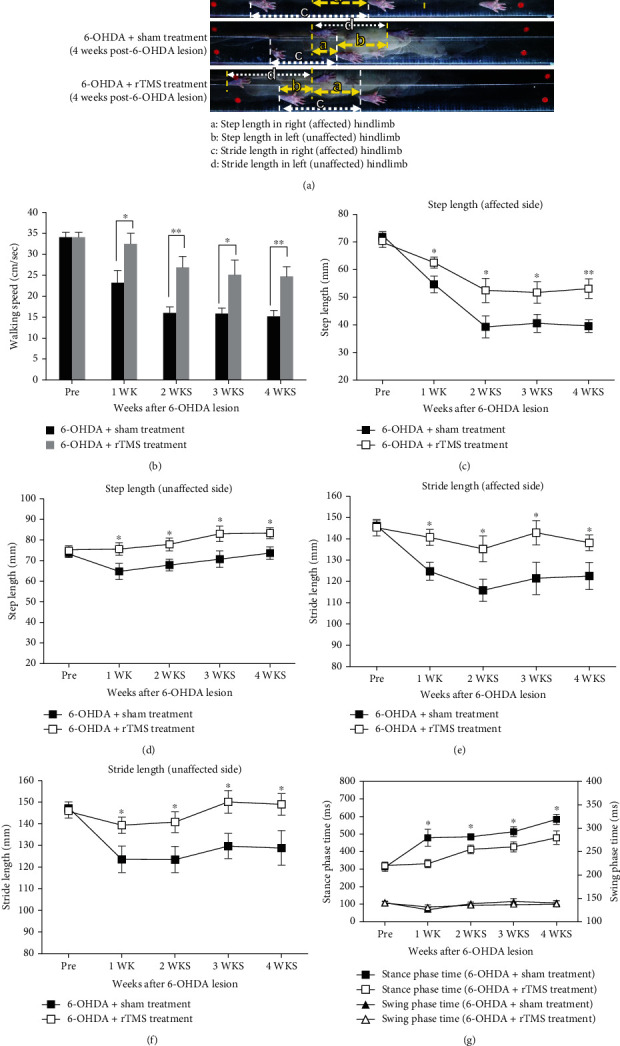
Characteristics of stepping footprint during 1 sec walkway locomotion in a rate pre-6-OHDA lesion, in a sham-treatment rat 4 weeks post-6-OHDA lesion, and in an rTMS-treated rat 4 weeks post-6-OHDA lesion (a). Time-course changes in walking speed (b), step length in the affected limb (c) and unaffected limb (d), stride length in the affected limb (e) and unaffected limb (f), and stance/swing phase time (g) in the sham- and rTMS-treated PD rats over 4 weeks of observation. Note that the step and stride lengths decreased significantly in the sham rTMS treatment group but decreased less in the rTMS treatment group. Gradual increases in the stance phases were found in both groups, but the sham treatment group showed a higher trend than the rTMS treatment group. No significant difference was found in swing phase duration. ^∗^*p* < 0.05 and ^∗∗^*p* < 0.01, significant post hoc Fisher's LSD differences when compared between the two groups at each time point.

For the results of gait analysis, Figure 4(a) shows typical footprint images recorded from a sham PD rat and real PD treatment rat in the early intervention group. Two-way repeated-measures ANOVA showed significant main effects of group in walking speed (*F*_1,24_ = 11.57, *p* = 0.002), step length (*F*_1,24_ = 8.84, *p* = 0.007 on the affected side; *F*_1,24_ = 11.93, *p* = 0.002 on the unaffected side), stride length (*F*_1,24_ = 15.56, *p* = 0.001 on the affected side; *F*_1,24_ = 13.46, *p* = 0.001 on the unaffected side), and stance phase time (*F*_1,24_ = 10.12, *p* = 0.004 on the affected side) but not in swing phase time (*F*_1,24_ = 0.13, *p* = 0.722 on the affected side), suggesting less impairment of gait pattern in the real treatment group than in the sham group. Subsequent post hoc *t*-tests between groups at each time point showed that this difference was largely driven by rTMS treatment effects observed starting at the first week of treatment on walking speed (*t* = 2.38, *p* = 0.025), step length (*t* = 2.16, *p* = 0.041 on the affected side; *t* = 2.18, *p* = 0.039 on the unaffected side), stride length (*t* = 2.81, *p* = 0.01 on the affected side; *t* = 2.20, *p* = 0.038 on the unaffected side), and stance phase time (*t* = 2.86, *p* = 0.009) (Figures 4(b)–4(g)). All these differences remained statistically significant at the end of the 4-week intervention (unpaired *t*-tests, *p* < 0.05).

### 3.2. Effects of rTMS Intervention Assessed by Immunohistochemistry

**Figure 5:**
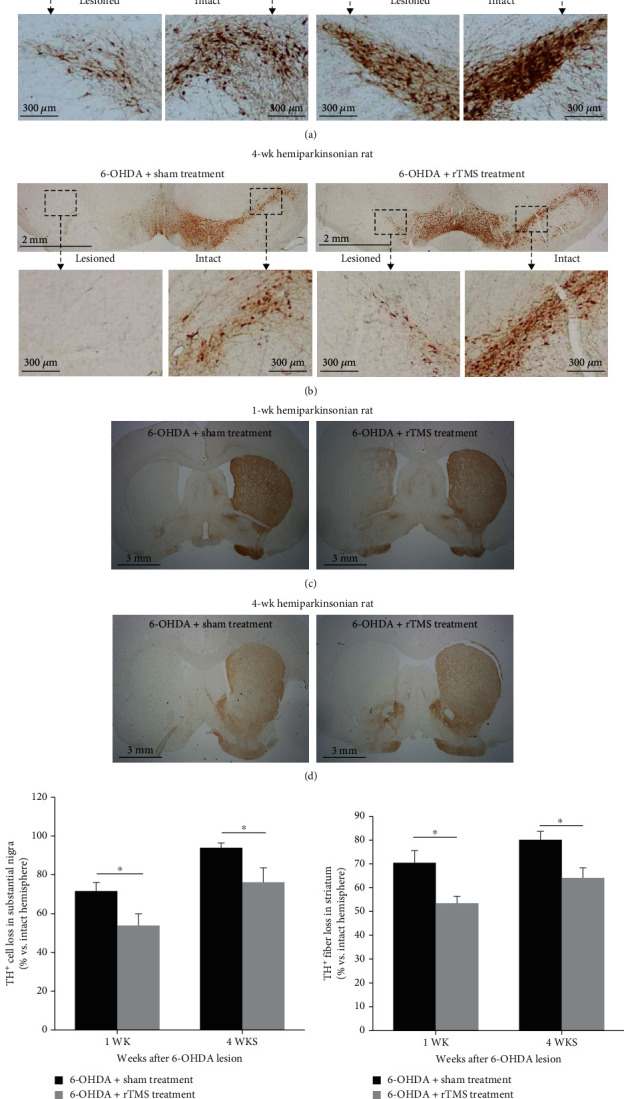
Representative TH-positive neurons in the substantia nigra pars compacta (a, b) and TH-positive fibers in the striatum (c, d) from sham control and rTMS-treated animals sacrificed at 1 week and 4 weeks post-6-OHDA lesion. Note the obvious reduction in TH-positive neurons or fibers in the lesioned hemisphere (left side) of the rTMS group at 1 week and 4 weeks post-6-OHDA lesion when the percentages of TH-positive neuron loss in the substantia nigra (e) and TH-positive fiber loss in the striatum (d) were compared to those in the sham treatment group. ^∗^*p* < 0.05 (unpaired *t*-test).

With regard to the effects of long-term rTMS intervention on dopaminergic neurons, the results of TH immunohistochemistry in the SNpc and striatum in rats at 1 wk and 4 wk post-6-OHDA lesion are shown in Figures 5(a)–5(d). The quantification of TH-positive cell loss in the substantia nigra and TH-positive fiber loss in the striatum at 1 week and 4 weeks post-6-OHDA lesion is presented in Figures 5(e) and 5(f). Rats that received rTMS intervention showed a preservation of TH-positive neurons in the SN (*t* = 2.338, *p* = 0.035 at week 1; *t* = 2.396, *p* = 0.031 at week 4) and TH-positive fibers in the striatum (*t* = 2.886, *p* = 0.012 at week 1; *t* = 2.837, *p* = 0.013 at week 4) compared to those that received sham stimulation.

## 4. Discussion

In the present study, we explored the hypothesis that long-term rTMS treatment with TBS would mitigate 6-OHDA-induced motor dysfunction and has a neuroprotective effect on dopamine neurons. We found that long-term rTMS intervention ameliorated progressive motor disturbances such as gait impairments (e.g., lower walking speed, shorter step/stride length, and longer stance phase time) and akinesia following 6-OHDA administration, indicating that early and long-term rTMS can suppress neurotoxin-induced motor impairments over repeated sessions of stimulation. Histological investigation revealed more preserved dopaminergic neurons in the SNpc and striatal fibers in the rTMS treatment group than in the sham group, suggesting a neuroprotective effect of rTMS.

Until now, the therapeutic efficacy and the detailed mechanisms of rTMS treatment for PD have remained inconsistent and unclear [33, 61–64]. For example, an improvement in motor symptoms in PD patients after rTMS treatment was reported, showing improvements in walking speed [65], Unified Parkinson's Disease Rating Scale part III (UPDRS-III) scores [66–71], 10 m walking test performance [68, 71], timed up-and-go test performance [69, 72], and freezing of gait (FOG) [69, 70, 73]. In contrast, some studies showed no significant improvement in rigidity, bradykinesia, tremor [74], functional performance of the hand [75], UPDRS-III scores [33, 76, 77], FOG [36, 78], gait, or bradykinesia [33, 77]. Although the exact underlying therapeutic mechanism is still unclear, the controversial results might be due to the variability of protocols, long-term pharmacological effects, clinical heterogeneity, and different severities of disease [37–40]. The use of an animal model could help in the control of confounding factors and may provide more information for clarifying the benefits of rTMS in PD and the underlying mechanisms its effects. Earlier animal studies have reported that rTMS treatment improved treadmill locomotor function and apomorphine/amphetamine-induced rotational behavior [43, 44]. In the present study, in addition to evaluations of rotational behavior, we performed comprehensive and quantitative assessments of gait and akinesia, which are the symptoms commonly observed in PD patients, to investigate the beneficial effects during and after four weeks of rTMS intervention. Moreover, our data show that long-term rTMS treatment has an accumulated effect on gait function. The time-course observation of such a comprehensive mean of behavioral tests is helpful for determining behavioral compensation and for quantifying the relative degeneration in dopaminergic neurons with disease progression. After hemiparkinsonian rats were treated with rTMS or sham treatment for four weeks, clear alleviation of gait dysfunction (e.g., lower walking speed, short step/stride length) was observed in the rTMS-treated group. When compared with the sham rTMS treatment group, we found that 4 weeks of rTMS treatment postponed disease progression after 6-OHDA injection. To the best of our knowledge, this is the first study confirming the therapeutic effects on gait disturbances and akinesia symptoms in hemiparkinsonian rats. This finding may also support clinical observations showing prolonged positive effects on gait function after rTMS treatment [40, 65, 79] and augments the growing amount of basic research and clinical literature on the efficacy of rTMS in PD treatment.

With regard to the effect of rTMS on akinesia, we found that four weeks of rTMS in hemiparkinsonian rats led to a reduction in akinesia. These results parallel PD human and animal studies showing a reduction in bradykinesia or forelimb akinesia after rTMS treatment, encouraging further research into the therapeutic potential of rTMS [79, 80]. The mechanisms by which rTMS improves several aspects of motor function in PD are still unclear. Evidence possibly supporting the efficacy of rTMS in PD is related to the release of dopamine induced by rTMS [81–84]. The widespread activation of dopaminergic neuronal systems or the elevation of serum dopamine concentration after repeated rTMS sessions could be one of the mechanisms for delaying the deterioration of motor dysfunction and may have contributed to the improvements in motor functions after daily sessions of rTMS observed in hemiparkinsonian rats [80, 85]. Another possible mechanism for the improvements in motor function following long-term rTMS treatment in PD could be related to the modulation of motor cortical plasticity induced by rTMS. In the classic complex cerebro-basal ganglia network, the usual facilitating effect of thalamic projections to the motor cortex is reduced in PD, resulting in the deactivation or hypoactivation of motor cortical areas and thus leading to reduced motor output during movement [86, 87]. Similar to the human TBS protocol, our earlier study indicated that rTMS with the iTBS protocol might be useful to promote motor cortical plasticity in healthy rats [45]. Furthermore, we previously demonstrated that rTMS-induced motor plasticity was reduced with time as the disease progressed in 6-OHDA-induced hemiparkinsonian rats, indicating that the change in motor plasticity is highly correlated with the degree of dopaminergic cell loss after PD lesions form [45]. Similarly, the impairments in the induction of the two forms of corticostriatal plasticity, long-term potentiation (LTP) and long-term depression (LTD), have been found to correlate with dopamine depletion and the onset of symptoms in the experimental parkinsonism rat model induced by 6-OHDA [88]. These impairments of bidirectional corticostriatal plasticity can be rescued by rTMS, which is linked to the increase in striatal dopamine levels produced by rTMS treatment [80]. Although we did not investigate the motor plasticity changes assessed by electrophysiological measures (e.g., motor evoked potential (MEP) elicited by TMS) in the current study, the possible mechanisms underlying the therapeutic effect in the improvement of motor functions could be via plasticity-like effects induced by long-term rTMS intervention in 6-OHDA-induced hemiparkinsonian rats when dopaminergic cells were preserved in the early stage after 6-OHDA injection.

Apomorphine-induced rotational behavior is a common method used to explore the level of dopamine depletion in 6-OHDA-induced hemiparkinsonian rats [45, 46, 89]. The time-course observations in the rotation test showed that the number of rotations observed in an hour gradually increased over the 4 weeks post-6-OHDA lesion in the sham-rTMS group, indicating a progressive increase in dopamine depletion with the increasing sensitization of the denervated dopaminergic receptors in the observation stage [89, 90]. However, the rotational response was reduced in the first and second weeks in rats that received rTMS treatment compared with rats that received sham treatment. These results indicated that early and intermittent high-frequency rTMS treatment could suppress the progression of dopamine depletion post-6-OHDA lesion over repeated sessions of stimulation. The effect of long-term rTMS treatment on the mitigation of 6-OHDA-induced progressive dopamine depletion in the early stage (i.e., 1-2 weeks post-PD lesion) was parallel with the histological observations, which showed neuroprotective effects against neurotoxin-induced damage to dopaminergic neurons and fibers in the SNpc and striatum, respectively. However, our findings revealed that 4 weeks of rTMS showed a neuroprotective effect in nigrostriatal dopaminergic neurons in histological analyses but not in rotational behavior tests since the rotation tests indicated that there were no differences between the rTMS and sham treatment groups in the number of rotations at weeks 3 and 4 post-6-OHDA lesion. No differences were observed between two groups in the number of rotations could be due to the dose of apomorphine for inducing spontaneous rotation being too high to show supersensitivity differences [91, 92]. In the current study, the dose of apomorphine was 0.5 mg/kg, a relatively high dose of apomorphine, which could easily lead to a plateau in rotational behavior [91]. To overcome this possible limitation, lower doses of apomorphine (0.05–0.1 mg/kg) may be more appropriate to detect the degree of dopamine receptor supersensitivity and maximize the difference between the rTMS and sham treatment groups [48, 91, 93].

The histological investigation showed that more dopaminergic cells survived in the group that received rTMS treatment than in the group that received sham rTMS stimulation. Such results suggest that daily rTMS intervention may not only improve motor functions but also have neuroprotective effects and mitigate the neurotoxin-induced damage to dopaminergic neurons. Similar neuroprotective results have been reported using low- (0.1 Hz) or high-frequency (10 Hz) rTMS [43, 44]. The neuroprotective effects on dopaminergic cells or fibers could be related to anti-inflammatory factors (e.g., cyclooxygenase-2 (COX-2) or tumor necrosis factor-alpha (TNF-*α*)); the upregulation of neurotrophic/growth factors such as brain-derived neurotrophic factor (BDNF), glial cell line-derived neurotrophic factor (GDNF), platelet-derived growth factor, and nerve growth factor (NGF); and a reduction in the astrogliosis and microglial activation (e.g., ionized calcium binding adaptor molecule 1 (Iba-1), glial fibrillary acidic protein (GFAP) induced by rTMS) [43, 44, 80]. Similar neuroprotective effects of rTMS were observed in brain injury and stroke animal models [94, 95]. With rTMS intervention using the TBS protocol, rTMS significantly reduced glial activation and neuronal death and improved functional recovery, indicating that rTMS may have potential as an antiapoptotic and anti-inflammatory treatment [94]. Furthermore, in an animal model of stroke, long-term rTMS induces complex changes in gene expression involved in angiogenesis, cellular repair, structural remodeling, neuroprotection, neurotransmission, and neuronal plasticity [95]. Although the underlying mechanisms are still unclear, further investigations are needed to clarify the detailed mechanisms underlying the neuroprotective effects of rTMS in PD.

## 5. Conclusion

In conclusion, the current study findings provide a clearer picture of progressive symptom changes with and without rTMS treatment and indicate the efficacy of rTMS in preventing motor and dopaminergic system abnormalities in a 6-OHDA hemiparkinsonian rat model. rTMS treatment improved motor functions and had a neuroprotective effect, showing that rTMS treatment preserved the function of dopamine neurons damaged by 6-OHDA administration in a rat model of PD. This long-term rTMS treatment model may serve as a bridge between animal and PD human studies. Future research is still needed to further clarify the underlying mechanisms and will lead to improved rTMS protocols and more effective PD therapies in humans.

## Data Availability

The data generated and analyzed during the current study are available from the corresponding authors on reasonable request.

## Abbreviations

6-OHDA: 6-Hydroxydopamine
BDNF: Brain-derived neurotrophic factor
BOS: Base of support
COX-2: Cyclooxygenase-2
DBS: Deep brain stimulation
GDNF: Glial cell line-derived neurotrophic factor
GFAP: Glial fibrillary acidic protein
Iba-1: Ionized calcium binding adaptor molecule 1
iTBS: Intermittent theta burst stimulation
NGF: Nerve growth factor
NHS: Normal horse serum
PBS: Phosphate-buffered saline
PD: Parkinson's disease
PFA: Paraformaldehyde solution
rTMS: Repetitive transcranial magnetic stimulation
SN: Substantia nigra
TBS: Theta burst stimulation
TH: Tyrosine hydroxylase.

## Supplementary Materials

Supplementary Materials Supplementary Table S1: the descriptive and inferential statistics of the primary outcomes for all the behavioral tests are shown.
