# Supplementary material for: Early Repetitive Transcranial Magnetic Stimulation Exerts Neuroprotective Effects and Improves Motor Functions in Hemiparkinsonian Rats
Source: Neural Plast. 2021 Dec 27;2021:1763533. doi: 10.1155/2021/1763533 (PMC8723880; doi:10.1155/2021/1763533)
Supplement: Supplementary Materials — Supplementary Table S1: the descriptive and inferential statistics of the primary outcomes for all the behavioral tests are shown. [file 1763533.f1.docx]

Supplementary Table S1: Descriptive and inferential statistics of the primary behavioral outcomes

|  | **6-OHDA + sham treatment group** | | | | | **6-OHDA + rTMS treatment group** | | | | | **Statistical analysis** | | |  |
| --- | --- | --- | --- | --- | --- | --- | --- | --- | --- | --- | --- | --- | --- | --- |
|  | Pre  PD lesion | 1 wk Post-  PD lesion | 2wks Post-  PD lesion | 3 wks Post-  PD lesion | 4 wks Post-  PD lesion | Pre  PD lesion | 1 wk Post-  PD lesion | 2wks Post-  PD lesion | 3 wks Post-  PD lesion | 4 wks Post-  PD lesion | Group | Time | Group*Time interaction |  |
| **Rotation behavior** | | - | 351±130* | 500±104* | 496±145 | 496±104 | - | 230±88* | 393±105* | 423±140 | 483±145 | P=0.023 | P<0.001 | P=0.255 |
| **Bar test for akinesia** | | 0.93±0.79 | 75.18±70.90* | 97.02±49.74* | 138.76±65.67* | 220.58±136.70* | 0.97±0.60 | 25.21±29.86* | 49.57±44.15* | 84.69±60.56* | 85.57±52.76* | P<0.001 | P<0.001 | P=0.003 |
| **Gait parameters** | |  |  |  |  |  |  |  |  |  |  |  |  |  |
| Walking speed | | 34.07±4.36 | 23.20±10.72* | 16.05±5.14* | 15.85±4.89* | 15.15±5.15* | 34.07±4.28 | 32.52±9.12* | 26.86±9.58* | 25.10±12.79* | 24.69±8.532* | P=0.013 | P<0.001 | P=0.002 |
| Step length (affected side) | | 72.33±4.97 | 54.65±11.00* | 39.31±14.28* | 40.56±11.60* | 39.58±8.12* | 69.90±6.72 | 62.52±7.22* | 52.48±15.75* | 51.74±13.94* | 53.11±12.86* | P=0.016 | P<0.001 | P=0.007 |
| Step length (unaffected side) | | 73.24±6.25 | 64.74±14.24* | 67.82±10.39* | 70.68±14.27* | 73.63±10.91* | 75.26±6.98 | 75.61±10.90* | 77.86±11.24* | 83.02±13.69* | 83.37±9.65* | P=0.386 | P=0.031 | P=0.002 |
| Stride length (affected side) | | 146.09±8.43 | 124.75±15.16* | 115.75±18.72* | 121.43±27.36* | 122.46±22.99* | 145.23±13.81 | 140.65±13.62* | 135.26±21.95* | 142.84±20.79* | 138.17±13.28* | P=0.181 | P=0.002 | P=0.001 |
| Stride length (unaffected side) | | 147.42±9.55 | 123.50±22.07* | 123.42±21.87* | 129.69±20.96* | 128.78±28.78* | 145.67±11.03 | 139.29±13.46* | 140.76±17.41* | 150.12±18.84* | 148.99±18.01* | P=0.156 | P=0.19 | P=0.001 |
| Swing phase time | | 140.83±11.49 | 125.03±16.23 | 138.38±13.84 | 143.40±22.06 | 140.39±16.52 | 139.80±11.21 | 131.50±16.67 | 135.17±11.92 | 136.52±17.68 | 137.86±25.19 | P=0.598 | P=0.026 | P=0.002 |
| Stance phase time | | 310.96±44.85 | 479.15±174.22* | 484.61±63.38* | 513.84±100.17* | 583.41±104.26* | 321.22±39.92 | 331.59±64.73* | 412.64±88.22* | 426.90±104.31* | 479.09±138.67* | P=0.025 | P<0.001 | P=0.004 |

All data are presented as mean ± SD. * Indicates a significantly difference between two groups (P < 0.05)
